# The Lubricating Effect of Eye Drops Containing Hyaluronic Acid and Mallow Extract in Patients with Dry Eye Disease—A Pilot Study

**DOI:** 10.3390/medicina59050958

**Published:** 2023-05-16

**Authors:** Andrea Attilio Basile, Giulia Mandelli, Magda Cendali, Rebecca Hufnagel

**Affiliations:** 1 Ophthalmological Practice, Via Andrea Solari 8, 20144 Milano, Lombardy, Italy; 2 Ophthalmological Practice, Via Gran San Bernardo 16, 20154 Milano, Lombardy, Italy; 3 Ophthalmological Practice, Via Palladio 26, 20135 Milano, Lombardy, Italy; 4 Clinical Research, Weleda AG, Moehlerstraße 3-5, 73525 Schwaebisch Gmuend, Baden-Wuerttemberg, Germany

**Keywords:** dry eye disease, hyaluronic acid, lubricating effect, mallow extract, medical device, pilot study, Visiodoron Malva^®^

## Abstract

*Background and Objectives*: Mucilaginous substances from plants are known to be able to support the lubricating effect of hyaluronic acid (HA) in dry eye disease (DED). In this pilot study, the combined lubricating effect of HA and mallow extract (*Malva sylvestris* L.) in patients with DED was assessed. *Materials and Methods*: Twenty patients at five ophthalmological practices in Italy were treated with eye drops containing HA and mallow extract on the one hand, and with eye drops containing HA only, on the other hand, in a two-period crossover design. As primary endpoints, the tear film breakup time (TBUT), the reduction of lissamine green staining of the ocular surface (Oxford Scheme, OS), and the safety and efficacy assessment by the ophthalmologists were evaluated. As secondary variables, the patient symptom score, the ocular surface index (OSDI) and the satisfaction, preference and efficacy assessment by the patients were evaluated. All data were analysed descriptively in addition to an exploratory analysis being made of the target variables. *Results*: Both products were well-tolerated. There were no statistically significant differences with regard to the TBUT, OS and OSDI between the two treatments. Anyway, the efficacy and safety assessments by the ophthalmologists and the patients showed results in favour of the combined product. *Conclusion*: The addition of mallow extract to HA-containing eye drops enhances the treatment of DED, at least with respect to subjective measurements. Further assessments will have to be done to prove and explain this observation in terms of measurable parameters, e.g., markers for inflammatory cytokines.

## 1. Introduction

Dry eye disease (DED) is one of the most frequently reported ophthalmologic diseases [1]. Andrew de Roetth introduced the term “dry eye” in 1950 [2]. Approximately 25% of the patients in ophthalmic clinics report symptoms of DED [3]. The prevalence ranges globally between 5% to 50% [4,5]. Women appear to be more affected by DED in all age groups. While former data suggested aging as one of the main risk factors [4], current data from a large Dutch cohort study show a relatively high prevalence in the age group of 20–30 years when it comes to symptomatic dry eye [4,5]. Between 3% (men) to 5% (women) of this age group reported “often” or “constant” symptoms of dry eye.

Dryness of the eyes, caused by a decreased aqueous phase of the tear film, was for a long time suspected to be the main characteristic of DED.

Nowadays, the definition according to the Tear Film & Ocular Surface Society’s Dry Eye Workshop II is: “Dry eye is a multifactorial disease of the ocular surface characterized by a loss of homeostasis of the tear film, and accompanied by ocular symptoms, in which tear film instability and hyperosmolarity, ocular surface inflammation and damage, and neurosensory abnormalities play etiological roles” [6].

DED can be caused by many different factors, e.g., intense screen work, drugs, hormonal changes, a variety of diseases, use of contact lenses, air pollution, air conditioners, as well as intense UV radiation [5,7,8,9,10]. Additionally, a genetic predisposition contributes to development of dry eye disease [11]. Typical reported symptoms are foreign body sensation, redness, itching and stinging, as well as burning in the eyes and transient visual disturbances [6,12,13].

In the treatment of DED, hyaluronic acid containing eye drops are well-described and established. Hyaluronic acid (HA), a compound found in the human body in greatest amounts in the skin, the synovial fluid, the vitreous body, and the umbilical cord, is characterized as well resorbable and biocompatible [14,15,16,17]. For instance, it is able to bind large numbers of water molecules. HA is known to play an important role in signal transduction, ovulation, fertilization, wound healing and tumour physiology [18]. Due to these properties, HA-containing treatments can be found in different medical fields. In ophthalmological treatments, HA is used as “lubricant” component as it shows great viscoelastic properties. It reduces the symptoms of DED by stabilising the tear film, reducing friction during blinking, and it prevents harmful substances from binding to the eye [19].

The medical device Visiodoron Malva^®^ is a novel composition of eye drops combining the beneficial effects of the established HA with a mucilaginous plant extract. The wild mallow was chosen because it contains plant mucus [20], which has the ability to bind and store water [21]. Mucilaginous substances from plants have the potential to support the lubricating effect of HA in DED [22,23]. This is suggested by a clinical study with an orally-taken food supplement containing a plant extract [22]. Also, a preclinical study in a murine model corroborates the beneficial effect of mucilaginous substances on DED [23].

During the development of this medical device, it was detected by physical measurement of the solution that the combination of HA and mallow extract resulted in a significantly reduced static surface tension compared to HA alone [24]. A lowered surface tension can ease spreading of the eye drops and assists in getting a good distribution of the solution onto the ocular surface. It can be concluded that the combined product remains longer on the eye and, theoretically, the eye is lubricated and refreshed for a longer period of time. To prove these observations in vivo, we conducted this pilot study, which compared the combined product with eye drops that only contained HA.

## 2. Materials and Methods

### 2.1. Study Design and Patient Population

This open-label, multicentre crossover pilot study was conducted at five ophthalmologic practices in Milan, Italy. Twenty patients were enrolled, four at each practice. All patients signed an informed consent form before study entry. The combination product as well as the comparator product are CE-marked medical devices and were applied according to their intended uses.

The study was conducted as a crossover study. Each patient administered each of the two eye drops products. It was up to the investigator to decide which of the two treatments was to be given first. Nine patients started with the combination of HA and mallow extract, and eleven patients received HA-only first. Treatment duration was 30 +/− 3 days for one product, followed by a washout phase of 7 days and another 30 +/− 3 days administration of the second product.

The patients (≥18 years), to be included, had to have a diagnostically confirmed moderate DED defined by TBUT < 10 s, OS > 2, and an OSDI between 23 and 32.

Patients underwent a general eye examination at baseline and periodically during the study. A history of eye infections or severe inflammation, as well as having had an eye surgery in the 6 months before the study were exclusion criteria. This was also the case for any uncontrolled serious systemic disease that could affect the eye, and other ongoing eye diseases.

### 2.2. Products Used, Dosage and Administration

Both products used were CE-certified medical devices. They were used in this study according to their intended uses. The first product, containing HA and mallow extract, was a sterile eye drop product in single doses (Visiodoron Malva^®^, Weleda AG, manufactured by Penta Arzneimittel GmbH, Stulln, Germany). A quantum of 1 mL of these eye drops contained 0.15% sodium hyaluronate, 0.5% extract of mallow flowers (*Malva sylvestris* L.), citrate buffer, sodium chloride and water for injection. The original package contained 20 single dose vials of 0.4 mL.

The second product, containing HA only, was also a sterile eye drop product in single doses (BLUyal^®^ UD, PHARMA STULLN GmbH, manufactured by Penta Arzneimittel GmbH, Stulln, Germany). A quantum of 1 mL of this comparator contained 0.15% sodium hyaluronate, sodium chloride, phosphate buffer and water for injection. The original package contained 20 single dose vials of 0.35 mL.

The HA used for both products had the same quality and was purchased from the same manufacturer. According to the certificates of analysis, both products were comparable with respect to their physical and chemical properties (e.g., in terms of pH, osmolality, and viscosity).

However, the surface tension of Visiodoron Malva^®^ was lower compared to that of the comparator BLUyal^®^ UD.

The patient instilled twice daily one drop in the lower conjunctival sac of each eye. For the treatment duration of 30 +/− 3 days, the participant used the same product and—after the washout period of 7 days—switched to the other product.

### 2.3. Efficacy and Safety Endpoints

As primary variables, the tear film breakup time (TBUT), the lissamine green staining of the ocular surface (Oxford Scheme, OS), and the efficacy and safety assessment by the ophthalmologist were assessed.

For evaluation of tear film stability, fluorescein was instilled into the eye and the interval between one complete blink and the first appearance of a dry spot in the precorneal tear film was measured in seconds with the slit lamp with cobalt blue filter. Values > 15 s are considered normal, values < 10 s are considered pathological.

Ocular surface staining is used as an indicator for the condition of the ocular surface. In this study, colour staining was used to detect epithelial defects of the ocular surface which could be attributed to inflammatory processes. For ocular surface colouring, lissamine green was used. The findings were graded according to the Oxford Scheme (OS) [25]. For each of the three zones (i.e., cornea, nasal bulbar conjunctiva and temporal bulbar conjunctiva,) a score from 1 to 3 was used. The maximum score (the worst case) was 9; the score is proportional to the dry eye clinical picture.

At the end of each treatment period, the ophthalmologist rated efficacy with the following categories: unsatisfactory, satisfactory, good and very good. Likewise, safety was rated using the same categories.

As secondary variables, intensity of symptoms (per eye, 7 patient-rated symptoms (each ranging from 0 (absent/none) to 10 (maximum intensity)), resulting in a total symptom score of 0 to 140), Schirmer test, ocular surface disease index (OSDI), efficacy assessment by the patient, patient satisfaction and individual patient preference were evaluated.

The OSDI is a scoring system consisting of three sub-scales, which in total cover 12 questions. The patient is asked to rate each symptom, condition, or situation from 0 (not present/none) to 4 (always). From the ratings, a sum-score is calculated. The resulting score is then classified according to a predefined grading system with the following categories: normal, mild, moderate and severe [26].

At the end of each treatment period, the patients rated efficacy using the following categories: 0 = no symptom relief, 1 = low symptom relief, 2 = good symptom relief and 3 = very good symptom relief. After each treatment period, the patients were asked to rate their satisfaction with the applied treatment according to the following categories: very unsatisfied, unsatisfied, satisfied, very satisfied, and I don’t know. After completion of both treatment periods, the patient was asked the following questions:

1. If you compare the first treatment period with the second one, have you perceived a difference?

Answer options: Yes (if yes, please specify), no, or I don’t know.

2. Which product would you prefer?

Answer options: Product 1, product 2, no preference, or I don’t know.

### 2.4. Statistical Analysis

A descriptive analysis of all data was performed, as well as an exploratory analysis of the target variables. For the analysis, the data were transferred from the Excel spreadsheets to SAS data sets using the SAS software version 9.3 (PROC IMPORT procedure). Five SAS data sets were created (visit 1, visit 2, visit 3, visit 4, and patient diary). The CRF and patient diary entries were checked for completeness and plausibility by the data management. Missing values were not tracked. The data of all patients for whom in each of both treatment periods at least the main target variable was documented at least once were included in the “intent-to-treat” (ITT) analysis. In the safety analysis, all patients were included who received and applied the medical device during the study.

## 3. Results

Between 16 December 2016 and 2 May 2017, 20 patients at five ophthalmologic practices in Italy were included in this study. All patients were included in the ITT-Set and the safety analysis set.

There were no premature terminations or withdrawals in this study.

### 3.1. Primary Variables

For both primary variables, namely, TBUT and OS, a dependency on the treatment sequence was analysed in order to detect carry-over effects. The analysis showed that a carry-over effect could be excluded (TBUT: *p* = 0.5804; OS: *p* = 0.2844). Consequently, data from both treatment periods was included in the analysis.

#### 3.1.1. Measurement of Tear Film Breakup Time (TBUT)

**Figure 1:**
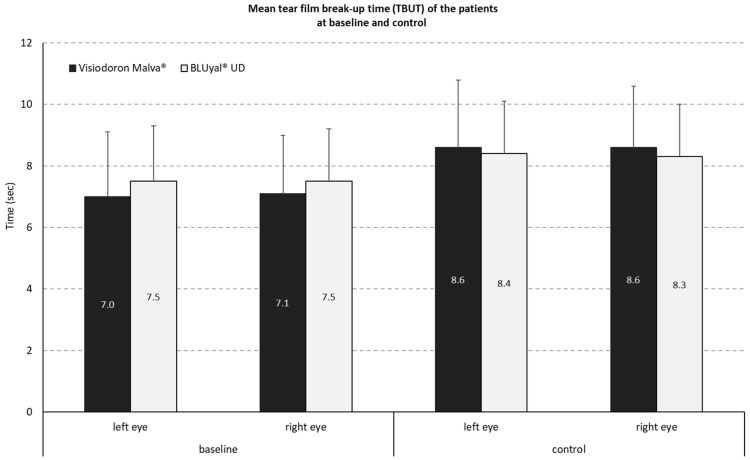
Mean tear film break-up time (TBUT) at baseline and control. The black panels show the values for the mallow extract containing eye drops, the white panels the ones of the comparator.

The distribution of TBUT values during the study showed, for the combined product, (containing HA and mallow extract) a mean difference of −1.5 s (SD 1.3 s) on the right eye and −1.6 s (SD 1.4 s) on the left eye. For the comparator preparation without mallow extract, the mean difference was −0.8 s, with a SD of 0.9 on the right eye and a SD of 1.0 s on the left eye (see Figure 1).

The analysis of the mean intra-individual differences of the mean TBUT reduction by a two-sided one sample t-test at an alpha level of 5% (with normal distribution assumption) resulted in a *p*-value of *p* = 0.1497. This means that there is no significant (5% level) difference in TBUT reduction between both treatments.

#### 3.1.2. Reduction of Lissamine Green Staining of the Ocular Surface (OS)

The mean OS reduction of both eyes was 1.13 for the eye drops containing HA in combination with mallow extract and 0.83 for HA alone. With respect to the condition of the ocular surface as graded by the OS, the positive reduction of mean OS values represents improvement for both treatments.

The analysis of the mean intra-individual differences of the mean OS reduction by a two-sided one-sample Wilcoxon signed-rank test at an alpha level of 5% (no normal distribution assumption) resulted in a *p*-value of *p* = 0.3149. This means that there is no significant (5% level) difference in OS reduction between the two products.

#### 3.1.3. Safety and Efficacy Assessments by the Ophthalmologists

**Figure 2:**
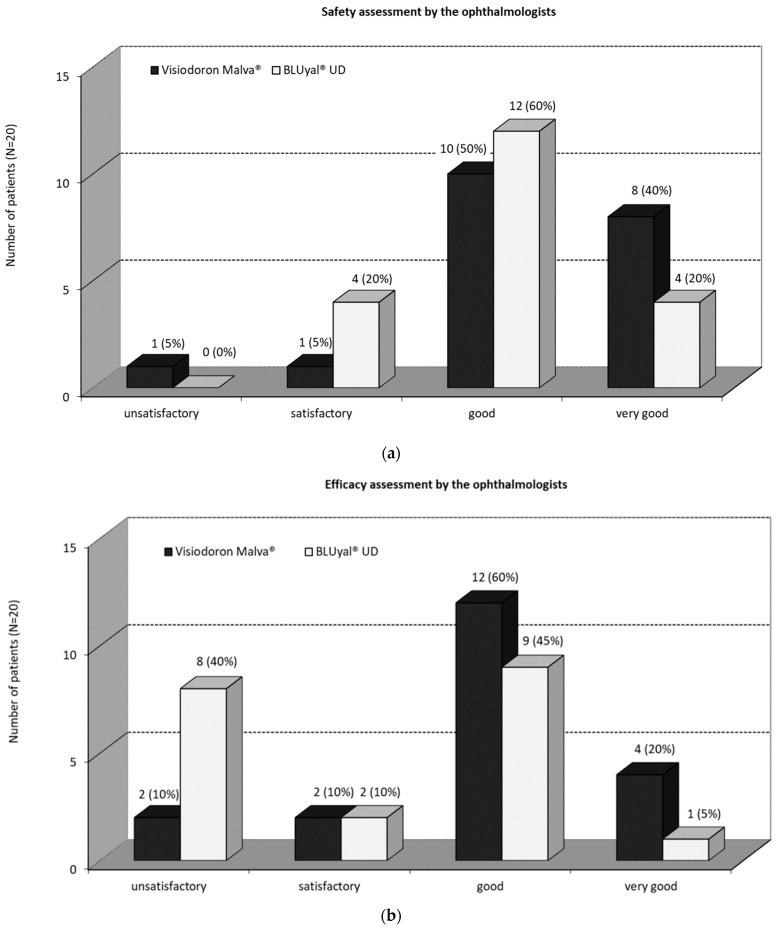
(**a**) Safety assessment by the ophthalmologists and (**b**) efficacy assessment by the ophthalmologists. The black panels show the values for the mallow extract containing eye drops, the white panels the ones of the comparator.

As one can see in Figure 2a, for ten patients (50%) the ophthalmologists rated the overall safety of the mallow extract containing medical device “good”, for eight patients (40%) “very good” and for one patient (5%) “satisfactory”. For twelve (60%) patients, the ophthalmologists rated the overall safety of the comparator as “good”, for four (20%) patients as “very good”, and for four (20%) patients as “satisfactory”. There was no significant difference (5% level) between the ratings (*p* = 0.2057, Fisher’s exact test). Anyway, the safety assessment by the ophthalmologists showed a better rating for the mallow extract containing eye drops, and the assessment “very good” was twice as high as that for the comparator product.

There was a recognizably better efficacy assessment by the ophthalmologists for the combination product compared to the eye drops only containing HA (Figure 2b). In the cases of four (20%) patients, the ophthalmologists assessed the overall efficacy of the mallow extract containing preparation as “very good”, for twelve (60%) patients as “good”, for two (10%) patients “satisfactory”, and for two (10%) patients “unsatisfactory”. The ophthalmologists rated the overall efficacy of the comparator treatment in one (5%) patient as “very good”, in nine (45%) patients as “good”, in two (10%) patients “satisfactory” and in eight (40%) patients “unsatisfactory”. The use of Fisher’s exact test (5% level) showed no significant difference (*p* = 0.1233).

### 3.2. Secondary Variables

#### 3.2.1. Total Symptom Score per Patient

**Figure 3:**
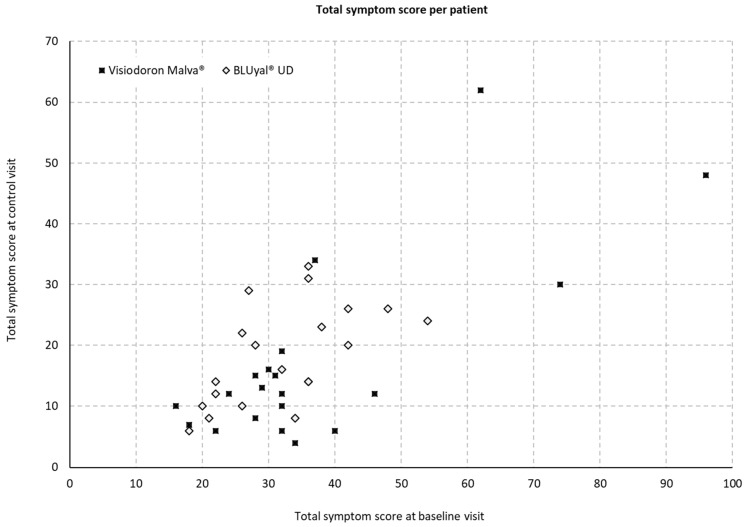
Total symptom score per patient = sum of scores for the symptoms: burning, itching, foreign body sensation, blurred vision, sensation of dryness, photophobia and pain; values were obtained for both eyes (score from 0 to 140).

At the baseline and the control visit, the following symptoms were assessed for both eyes: burning, itching, foreign body sensation, blurred vision, sensation of dryness, photophobia and pain. Figure 3 displays the total symptom scores on both visits and for both treatments. The scoring can range from 0 to 140.

The evaluation of the sum-score of all symptoms showed a mean difference of 9.95 with an SD of 6.39 (minimum 0, maximum 24 and median 8) for the eye drops containing HA and mallow extract and 6.95 with an SD of 4.19 (minimum −1, maximum 15 and median 7) for the comparator. However, a two-sample t-test with a sufficient normal approximation at alpha level of 5% showed no significant difference for the alleviation of symptoms between the two medical devices (*p* = 0.0873).

#### 3.2.2. Ocular Surface Disease Index (OSDI)

**Figure 4:**
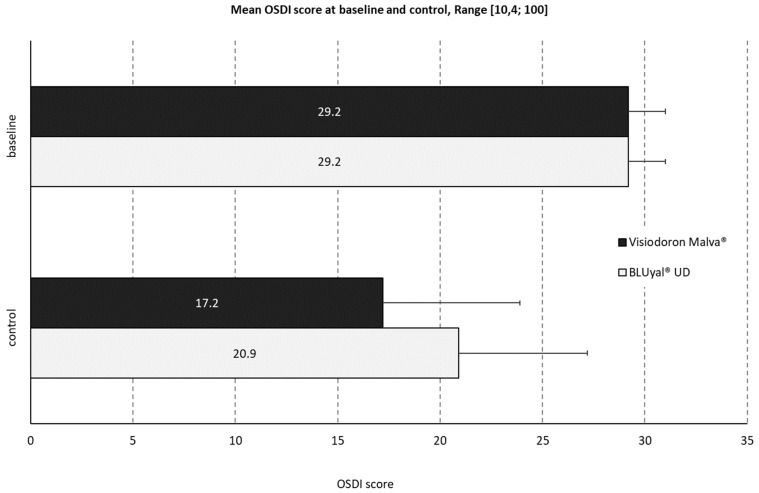
Mean ocular surface disease index (OSDI) score at baseline and control. The black panels show the values for the mallow-extract-containing eye drops, the white panels the ones of the comparator.

The mean baseline OSDI score (29.2, SD 1.8) was identical for both products (see Figure 4). The control score of the mallow-extract-containing preparation was 17.2 (SD 6.7, minimum 8.3, maximum 35.0 and median 16.7). For the comparator, the mean OSDI control score was slightly higher: 20.9 (SD 6.3, minimum 10.4, maximum 33.3 and median 19.8).

At baseline, all patients had a ‘moderate’ OSDI classification. After treatment with the product containing HA and mallow extract, 90% of the patients improved to a ‘mild’ or ‘normal’ classification. For the comparator product, only 60% of the patients showed an improvement in classification.

For both treatments, the mean differences of the Schirmer test values (baseline minus control) were within the same range. 

#### 3.2.3. Assessment by the Patients

**Figure 5:**
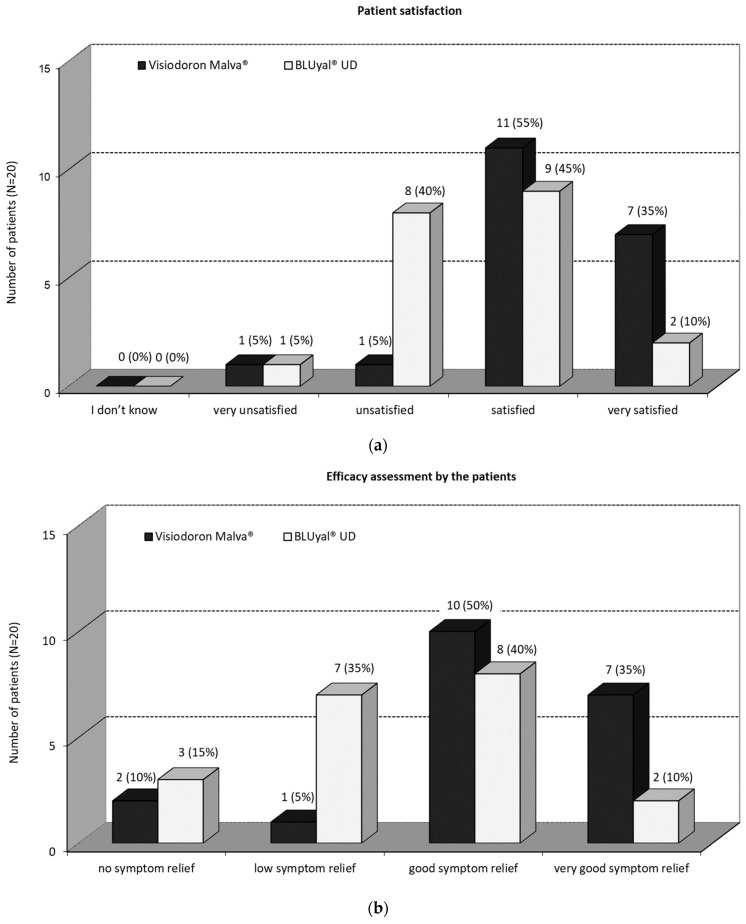
(**a**) Patient satisfaction and (**b**) efficacy assessment by the patients. The black panels show the values for the mallow-extract-containing eye drops, the white panels the ones of the comparator.

As one can see from Figure 5a, a great majority of the patients were satisfied or very satisfied with the eye drops containing mallow extract. With the treatment of the comparator product, 45% of the patients were satisfied, 10% were very satisfied, 40% were unsatisfied and 5% were very unsatisfied. There was a significant difference (5% level) in favour of the mallow-extract-containing eye drops (*p* = 0.0226; Fisher’s exact test).

Figure 5b shows that 85% of the patients assessed their symptom relief as good or even very good after treatment with the HA and mallow-extract-containing preparation. For the HA eye drops without mallow extract, 40% of the patients reported a good symptom relief, 10% a very good symptom relief, 35% a low symptom relief and 15% had no symptom relief. There was a significant difference (5% level) in favour of the combination product (*p* = 0.0503; Fisher’s exact test).

In total, the efficacy assessment by the patients as well as the patient satisfaction shows a trend in favour of the eye drops containing mallow extract.

#### 3.2.4. Individual Patient Preference

**Figure 6:**
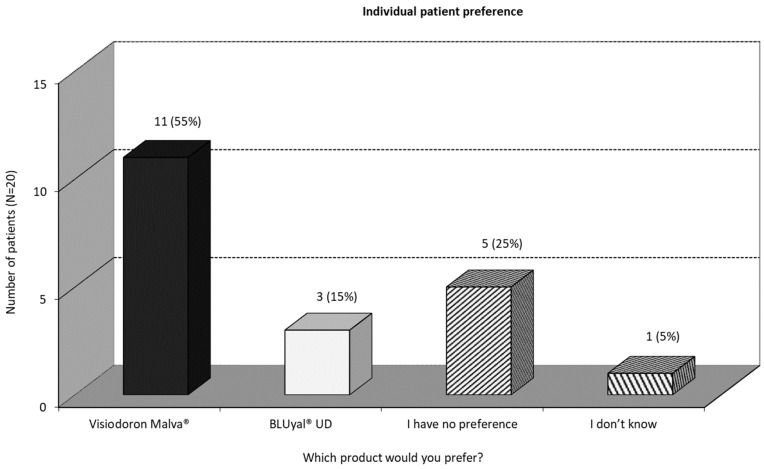
Individual patient preference.

At the end of the study, all patients were asked for their preferred treatment. As one can see in Figure 6, more than half of the patients indicated a preference for the mallow-extract-containing preparation, 25% had no preference and 15% indicated a preference for the eye drops containing only HA.

One can summarize, that, in terms of subjective measurement, the eye drops containing mallow extract are the preferred treatment according to this pilot study.

#### 3.2.5. Safety Assessment

There were no incidents. Consequently, no adverse events (AEs) or serious adverse events (SAEs) were reported or documented during the study.

## 4. Discussion

Artificial tears for topical application are the established treatment for alleviating the symptoms of DED [27]. There is a wide range of ocular lubricants with different properties available [28,29,30,31,32]. In terms of efficacy, patients seem to benefit from the treatment, but the available clinical evidence is not well-defined and needs to be supported by further clinical studies. The treatments’ safety is generally good, however some patients report side effects, such as blurred vision. The motivation for this exploratory pilot study was to generate initial data that allows assessment of the combined lubricating effect of HA and mallow extract.

As demonstrated from the results, both treatments were associated with improvements in patients with moderate DED. This is in line with expectations, as both products contain HA, which effectively lubricates the ocular surface and thereby alleviates symptoms.

For the measured efficacy parameters TBUT, OS-grading and OSDI, and also for the symptoms assessed during the visits at the ophthalmologic practices, no significant differences between the mallow-extract-containing preparation and its comparator were observed.

On the other hand, there were recognizable and, in parts, statistically significant differences between the treatments regarding patient satisfaction, individual patient preference and assessments of efficacy by patients and ophthalmologists. There was a difference in alleviation of symptoms as well as patient satisfaction and individual patient preference in favour of the eye drops containing HA in combination with mallow extract.

Both products were well tolerated, and no incidents were reported. Both preparations can be assessed as safe.

To the authors’ knowledge, and after a literature search in several databases, there are no other products on the market for topical application in humans that combine HA with mucilaginous substances. According to the literature, comparison of different artificial tears has not shown a clear advantage of a particular class of ocular lubricants [33]. Consequently, at this stage, a comparison of the present study results with other products is not possible.

To what extent the observed differences between the mallow-extract-containing preparation and its comparator can be attributed to the mallow extract remains unclear, as the explorative design of this pilot study by its nature can only provide indications and deliver qualitative answers to this question. Anyway, earlier studies have shown that plant extracts improve tear film stability [22,23]. Also, the antioxidant activity [34] and anti-inflammatory effects of mallow [35] are described in the literature. All of these effects can influence lacrimal glands and membrane cells, thereby delivering treatment options in DED.

## 5. Conclusions

DED is a common disease with a variety of symptoms that affect patients’ quality of life. Treatment with HA-containing eye drops is established. As shown in this clinical pilot study, both treatments resulted in improvements in patients with moderate DED. However, the combination of mallow extract with HA was perceived as superior by patients, as compared to eye drops containing HA alone. This indicates that mallow extract contributes positively to the overall efficacy of Visiodoron Malva^®^. Reduction of surface tension caused by mallow extract could provide a possible explanation for this perceived superiority. The distribution of the eye drops on the ocular surface may thus be improved, possibly resulting in a prolonged moisturization of the eye.

Further investigations are needed to assess the impact of mallow extract on treatment of DED, for example, measurements of the osmolarity of the tear film or with markers for inflammatory cytokines.

## Data Availability

The data that support the findings of this study are not publicly available due to the fact that they contain information that could compromise the privacy of research participants. Disclosure of data would not be in accordance with the European General Data Protection Regulation.
